# A Case of Vaginal Birth after Cesarean Delivery in a Patient with Uterine Didelphys

**DOI:** 10.1155/2019/3979581

**Published:** 2019-12-23

**Authors:** Jennifer W. H. Wong

**Affiliations:** Department of Obstetrics, Gynecology and Women's Health, John A. Burns School of Medicine, University of Hawaii, Honolulu, Hawaii, USA

## Abstract

**Background:**

The American College of Obstetricians and Gynecologists (ACOG) recommends that most women with one prior low-transverse cesarean delivery should be offered a trial of labor after cesarean (TOLAC). However, very little is known about TOLAC in women with uterine anomalies.

**Case:**

A 32-year-old gravida-2 para-1 female with a history of uterine didelphys and one prior low-transverse cesarean section in the left uterine horn presented with a subsequent pregnancy in the left uterine horn. After extensive counseling on TOLAC versus repeat cesarean delivery, the patient decided to proceed with TOLAC and had a spontaneous vaginal delivery of a healthy infant at 38 3/7 weeks of gestation.

**Conclusion:**

TOLAC can be considered in women with uterine anomalies using ACOG's standard TOLAC guidelines with informed consent and shared decision-making between the patient and obstetrician.

## 1. Introduction

The rate of cesarean delivery (CD) in the United States has increased more than six-fold between the years 1970 and 2016, with the most recent rate reported to be 31.9% [1]. Promoting trial of labor after cesarean (TOLAC) among appropriate candidates safely decreases the nation's CD rate, in addition to preventing surgical complications and future pregnancy complications associated with CD. The American College of Obstetricians and Gynecologists (ACOG) recommends that “most women with one previous cesarean delivery with a low-transverse incision are candidates for and should be counseled about and offered TOLAC” [2]. This level A recommendation is based on good and consistent scientific evidence.

However, little is known about TOLAC in women with uterine anomalies. Given the rarity of this situation, ACOG does not offer practice guidelines for TOLAC in women with uterine anomalies [2]. In 2015, *Obstetrics & Gynecology* published a case report of a woman with a history of CD in the right horn of her didelphic uterus, followed by a vacuum-assisted vaginal delivery of a subsequent pregnancy in her contralateral left horn [3]. In contrast, we report a high-risk case of a woman with a history of CD in the left horn of her didelphic uterus, followed by a spontaneous vaginal delivery of a subsequent pregnancy in the same left uterine horn.

## 2. Case Presentation

A 32-year-old gravida-2 para-1 female at 12 1/7 weeks of gestation presented to the office to establish prenatal care. The patient had a history of known uterine didelphys, with confirmation of two uteri, two cervixes, a vertical vaginal septum, and a normal renal system on magnetic resonance imaging. Her obstetrical history was significant for a prior low-transverse CD at 35 1/7 weeks for preterm labor with a fetus in the breech presentation in the left uterine horn three years prior. The current pregnancy was also located in the left horn. The patient declined cervical length screening and 17-alpha hydroxyprogesterone caproate for her history of preterm labor. She had an otherwise uncomplicated pregnancy, and at 32 0/7 weeks of gestation, a growth ultrasound showed that the fetus was growing well at the 51^st^ percentile for weight. She was extensively counseled throughout her pregnancy on TOLAC versus repeat CD, and the patient decided to proceed with TOLAC.

At 38 3/7 weeks of gestation, the patient presented to labor and delivery for early labor with spontaneous rupture of membranes. An epidural was received for analgesia. Labor was augmented with intravenous oxytocin and progressed well. During the second stage of labor, the patient's vaginal septum was manually displaced while pushing, and less than one hour later, the patient had a spontaneous vaginal delivery of a healthy female infant. The vaginal septum was noted to have torn during delivery, so the remainder of the septum was transected, and the incised edge was closed with polyglycolic acid suture in a running fashion for hemostasis. Estimated blood loss was 300 mL. The patient's postpartum course was uncomplicated, and she was discharged home on postpartum day 2.

## 3. Discussion

To the best of our knowledge, this is the first-ever published case of a successful vaginal birth after cesarean (VBAC) in a woman with uterine didelphys and a history of prior CD with a subsequent pregnancy in the same uterine horn. Although data are limited, TOLAC can be considered in women with uterine anomalies using ACOG's standard TOLAC guidelines with informed consent and shared decision-making between the patient and obstetrician.

Uterine anomalies are associated with multiple obstetric complications, including recurrent early pregnancy loss [4], preterm birth [4–6], preterm prelabor rupture of membranes [5], malpresentation [4–6], low birth weight [6], intrauterine fetal demise [5], and cesarean delivery [5, 6]. Given these risks, the fetus should be monitored with serial growth ultrasounds [4]. Consultation with maternal-fetal medicine and routine antepartum testing can also be considered. Counseling on mode of delivery should be initiated early in pregnancy.

Little is known about TOLAC in women with uterine anomalies, and only a few studies exist in the medical literature. A retrospective population-based study of 165 women with uterine anomalies found that these women were statistically less likely to have a VBAC than women with normal uteri (37.6% vs. 50.7%, *P* < 0.001) and that they were less likely to experience uterine rupture than women with normal uteri (0% vs. 0.002%) [7]. Another smaller study comprised of 25 women with uterine anomalies reported a VBAC rate similar to that in women with normal uteri (80.0% vs. 74.9%) and a uterine rupture rate significantly higher than that in women with normal uteri (8.0% vs. 0.6%, *P* = 0.01) [8]. These studies are limited by sample size but nonetheless provide important information about an understudied area of obstetrics. While attempting TOLAC in women with uterine anomalies appears to be a relatively successful and safe way to decrease the rate of CDs, additional studies are needed to determine the true incidence of VBAC and uterine rupture.

The actual rates of VBAC and uterine rupture in women with uterine anomalies are likely to be similar to or less favorable than those in women with normal uteri. Some authors hypothesize that uterine anomalies, especially unicornuate uteri, are associated with decreased uterine muscle mass [9, 10]. If this hypothesis was true, we can hypothesize that decreased uterine muscle mass may result in inadequate uterine contractions and arrest disorders of labor, thereby increasing the rate of operative vaginal delivery and repeat CD. Provider bias may also contribute to the increased number of repeat CDs. Providers may have a lower threshold to withhold oxytocin or repeat CD given the unknown risk of uterine rupture. If uterine anomalies have decreased muscle mass, we can also hypothesize that an associated decrease in vascularity could impair scar formation and increase the risk of uterine rupture. These are all hypotheses that need to be further investigated. Given the limited data available, informed consent and shared decision-making between the patient and obstetrician are essential when considering TOLAC in women with uterine malformations.

## Abbreviations

CD: Cesarean delivery
TOLAC: Trial of labor after cesarean
ACOG: American College of Obstetricians and Gynecologists
VBAC: Vaginal birth after cesarean.
